# Efficacy and Safety of Low-Dose Bisoprolol/Hydrochlorothiazide Combination for the Treatment of Hypertension: A Systematic Review and Meta-Analysis

**DOI:** 10.3390/jcm13154572

**Published:** 2024-08-05

**Authors:** Arrigo F. G. Cicero, Naif Saad ALGhasab, Giuliano Tocci, Giovambattista Desideri, Giulia Fiorini, Federica Fogacci

**Affiliations:** 1 Hypertension and Cardiovascular Risk Factors Research Centre, Medical and Surgical Sciences Department, Alma Mater Studiorum University of Bologna, 40100 Bologna, Italy; 2 Cardiovascular Medicine Unit, IRCCS AOU BO, 40100 Bologna, Italy; 3 Department of Cardiology, Libin Cardiovascular Institute, Calgary University, Calgary, AB T2N 1N4, Canada; dr.naif.saad@gmail.com; 4 Department of Internal Medicine, Medical College, Ha’il University, Ha’il 55476, Saudi Arabia; 5 Cardiology Unit, Division of Cardiology, Department of Clinical and Molecular Medicine, Faculty of Medicine and Psychology, University of Rome ‘La Sapienza’, Sant’Andrea Hospital, 00154 Rome, Italy; giuliano.tocci@uniroma1.it; 6 Department of Clinical, Internal, Anesthesiologic and Cardiovascular Sciences, Sapienza University of Rome, 00161 Rome, Italy; giovambattista.desideri@uniroma1.it

**Keywords:** hypertension, blood pressure, bisoprolol, hydrochlorothiazide, meta-analysis

## Abstract

**Objectives:** This systematic review and meta-analysis aimed to assess the blood pressure (BP)-lowering effect and the safety profile of low-dose bisoprolol/hydrochlorothiazide combination treatment in patients with hypertension. **Methods:** Multiple electronic databases were systematically searched, and five clinical studies were included in the meta-analysis. **Results:** Treatment with bisoprolol/hydrochlorothiazide significantly reduced systolic BP (SBP) [mean difference (MD): −8.35 mmHg, 95% confidence interval (CI): −11.44, −5.25 mmHg versus control; MD: −9.88 mmHg, 95%CI: −12.62, −7.14 mmHg versus placebo] and diastolic BP (DBP) [MD: −7.62 mmHg, 95%CI: −11.20, −4.04 mmHg, versus control; MD: −8.79 mmHg, 95%CI: −11.92, −5.67 mmHg versus placebo]. Moreover, BP response rate and BP control rate after low-dose bisoprolol/hydrochlorothiazide combination treatment were significantly greater compared to control [odd ratio (OR) for response rate: 4.86, 95%CI: 2.52, 9.37; OR for control rate: 1.67, 95%CI: 1.11, 2.51]. Finally, treatment with low-dose bisoprolol/hydrochlorothiazide was associated with a reduced risk of any adverse event (AE) and peripheral edema compared to control. **Conclusions:** Overall, our results reaffirm the safety and efficiency of prescribing bisoprolol/hydrochlorothiazide combination treatment in stage I and II hypertension.

## 1. Introduction

Hypertension is a leading risk factor not only for stroke and coronary heart disease but also for chronic kidney disease and dementia [1,2,3,4]. The global trend of hypertension showed a twofold increase in the absolute number of patients with hypertension over the course of three decades until 2019 [1]. The dramatic effects of this significant worldwide trend are partially compensated by the availability of some major classes of effective anti-hypertensive drugs, such as calcium channel blockers, renin-angiotensin system (ACE) inhibitors/angiotensin receptor blockers (ARBs), beta-blockers, and diuretics [5].

Thiazide diuretics, namely chlorthalidone (a thiazide-like diuretic) and hydrochlorothiazide (a thiazide-type diuretic), have been the mainstay of blood pressure (BP) management since their discovery over half a century ago [5,6]. Beta-blockers have been recognized as a prominent and effective initial therapy for hypertension since the late 1960s, as they exert their therapeutic effects by targeting the sympathetic excitation pathway [5,7]. However, before the most recent international guidelines of the European Society of Hypertension (ESH) and the European Society of Cardiology (ESC) re-evaluated the use of beta-blockers as first-line anti-hypertensive agents [8], they were less commonly prescribed, being favored with more potent vasodilators that prevent cardiac outcomes, such as stroke and heart attack [9,10]. In effect, beta-blockers may not lower central BP as effectively as other medications [11]. They exert their effects by slowing down the heart rate, which can prolong the ejection period. This prolongation may in turn increase systolic BP (SBP) when beta-adrenoceptor blockers (beta-blockers) are used alone [12]. However, most patients with hypertension require a combination of BP-lowering agents to achieve targeted BP control [13,14,15]. The 2021 World Health Organization (WHO) hypertension guidelines recommend the inclusion of a fixed-dose combination of two BP-lowering medications. These combinations are also incorporated into the 22nd WHO Model List of Essential Medicines, which not only includes multiple combinations but also up to four specifically tailored for hypertension [16]. A combination of multiple medications can target different mechanisms for synergistic effects and better blood pressure control, leading to improved clinical outcomes [17].

This systematic review and meta-analysis aimed to evaluate the efficacy and safety of bisoprolol/hydrochlorothiazide combination therapy for BP control in patients with stage I and II hypertension. The primary outcome of this study was to assess the BP-lowering effect of bisoprolol and hydrochlorothiazide in patients with hypertension. The secondary outcome was to assess the clinical safety of bisoprolol and hydrochlorothiazide in the treatment of hypertension.

## 2. Methods

### 2.1. Study Design

This study was designed in accordance with the guidelines of the 2020 Preferred Reporting Items for Systematic Reviews and Meta-Analysis (PRISMA) statement [18], and its protocol was prospectively registered in PROSPERO (reference number: CRD42024517663). Owing to the study design (i.e., meta-analysis), neither Institutional Review Board (IRB) approval nor patient informed consent was required.

### 2.2. Search Strategy

This study is based on an extensive literature search conducted in PubMed Medline, SCOPUS, Google Scholar, and Web of Science by Clarivate, with no language restriction, using the following search terms: “Bisoprolol” AND “Hydrochlorothiazide” AND (“Clinical trial” OR “Clinical study” OR “Randomized” OR “RCT” OR “Double-blind” OR “Single-blind”) AND (“Hypertension” OR “Blood pressure” OR “BP”). The wild-card term “*” was used to increase the sensitivity of the search strategy, which was limited to studies in humans. The reference lists of the identified papers were manually checked for additional relevant articles. Additional searches for potential trials included references of review articles in that issue and abstracts from selected congresses. A literature search was conducted from its inception to 5 November 2022.

All abstracts were screened to exclude ineligible articles. The full text of the remaining articles was obtained and assessed again, evaluating each article independently and performing data extraction and quality assessment.

### 2.3. Study Selection Criteria

Studies were included if they met the following criteria: (i) being randomized clinical trials with either parallel or crossover design, (ii) having appropriate controlled designs, and (iii) investigating the effect of bisoprolol and hydrochlorothiazide combination treatment on BP.

The exclusion criteria were as follows: (i) lack of randomization for treatment allocation, (ii) lack of blindness, and (iii) lack of a control group receiving placebo or a BP-lowering control treatment different from bisoprolol and hydrochlorothiazide. Studies were also excluded if they enrolled individuals overlapping with other studies.

### 2.4. Data Extraction

Data extracted from the eligible studies were as follows: (i) first author’s name, (ii) year of publication, (iii) study location, (iv) follow-up, (v) main inclusion criteria and underlying disease, (vi) tested intervention, (vii) study groups, (viii) number of participants in the active and control groups, (ix) age of study participants, and (x) baseline and outcome data of SBP and diastolic BP (DBP). Safety outcomes included adverse events (AE) such as headache, insomnia, dizziness, fatigue, bradycardia, hypokalemia, cough, dyspnea, nausea, diarrhea, peripheral edema, decrease or loss of libido, and impotence. All verbatim terms for adverse events were coded to the preferred term and system-organ class using the Medical Dictionary of Regulatory Activities, maintaining the classification originally performed in individual clinical trials.

Missing or unpublished data were sought by contacting the authors via email, and repeated messages were sent in case of no response.

### 2.5. Risk of Bias Assessment

The risk of bias in the included randomized controlled clinical studies was systematically assessed using the version 2 of the Cochrane risk-of-bias tool for randomized trials (RoB-2 tool) that took into account the following domains: randomization, deviations from intended interventions, missing outcome data, and measurement of the outcome and selection of the reported results [19]. Two authors independently performed the risk-of-bias assessment [20]. Disagreements were resolved by consensually agreed principles.

### 2.6. Data Synthesis

Meta-analysis was conducted using Comprehensive Meta-Analysis (CMA) Version 3 software (Biostat, NJ, USA) [21].

The effect sizes for changes in SBP and DBP were expressed as mean differences (MD) and 95% confidence intervals (CI). The net changes in the investigated parameters (change scores) were calculated by subtracting the value at baseline from that after the intervention in the active-treated and control groups. Standard deviations (SDs) of the MDs were obtained by following the method described by Follman et al., assuming a correlation coefficient (R) = 0.5 [22]. For safety analysis, odds ratios (OR) and 95% CI intervals were calculated using the Mantel–Haenszel method. If one or more outcomes could not be extracted from the study, it was removed only from the analysis involving those outcomes. Adverse events were considered for analysis only if they occurred in at least two of the included clinical trials.

The level of inter-study heterogeneity was quantitatively assessed using the Higgins index (I^2^) [23]. Possible sources of heterogeneity were explored through sub-group analyses by control treatment [24]. The studies’ findings were combined using a random-effect model to provide a more conservative assessment of the precision of the summary estimate [25]. Sensitivity analysis was conducted to account for the risk of bias and evaluate the robustness of the main findings. A leave-one-out method was used (i.e., one study was removed at a time, and the analysis was repeated) [26].

Two-sided *p*-value < 0.05 was considered statistically significant.

### 2.7. Publication Biases

Potential publication biases were explored using the visual inspection of Begg’s funnel plot asymmetry, Begg’s rank correlation test, and Egger’s weighted regression test [27]. The Duval and Tweedie “trim and fill” method was used to adjust the analysis for the effects of publication biases [28]. In the case of a significant result, the number of potentially missing studies required to make the *p*-value non-significant was estimated using the classical fail-safe ‘N’ method as another marker of publication bias. Two-sided *p*-values < 0.05 were considered statistically significant.

## 3. Results

**Figure 1:**
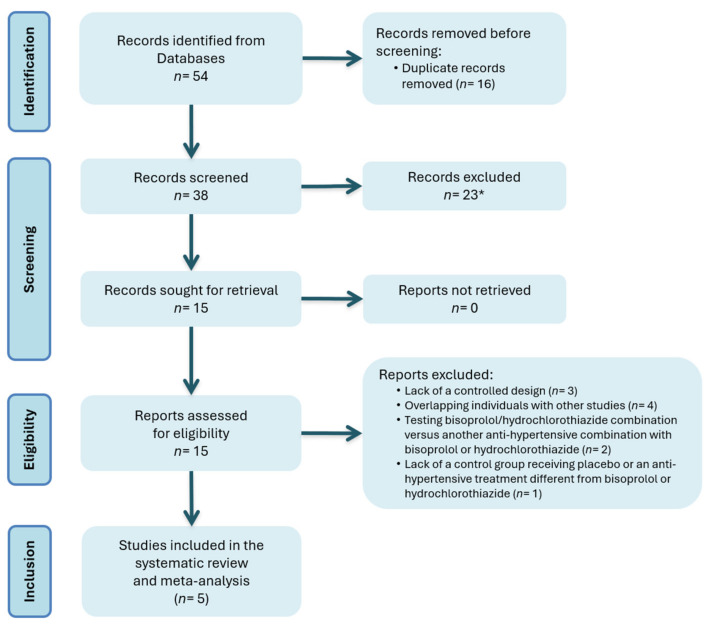
Flow chart of the literature search. * Twenty-three records screened were excluded after abstract reviewing as data were not consistent with search criteria.

After database searches and an assessment of eligible studies, five articles were included in the meta-analysis. The study selection process is shown in Figure 1 (more details have been provided in Appendix A).

Eligible studies were published between 1993 and 2002 and conducted in the United States of America (*n* = 5) and France (*n* = 1). The follow-up period ranged from 4 to 17 weeks.

The main characteristics of the studies included in this meta-analysis are summarized in Table 1.

### 3.1. Risk of Bias Assessment

Almost all the studies included in the meta-analysis reported sufficient information regarding allocation concealment, sequence generation, and personal and outcome assessments. More details on the quality of the bias assessment are provided in Table 2.

### 3.2. Bisoprolol/Hydrochlorothiazide Effect on SBP

**Figure 2:**
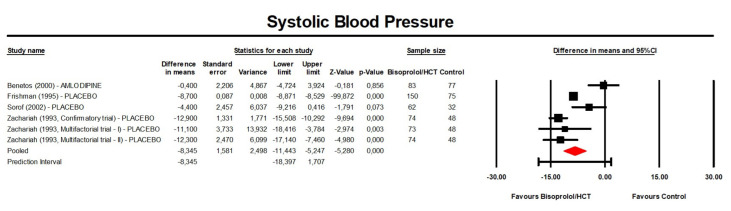
Forest plot displaying mean differences and 95% confidence intervals (CI) for the effect of bisoprolol/hydrochlorothiazide on systolic blood pressure compared to the control (placebo or another anti-hypertensive treatment). CI, confidence interval; HCT, hydrochlorothiazide.

**Figure 3:**
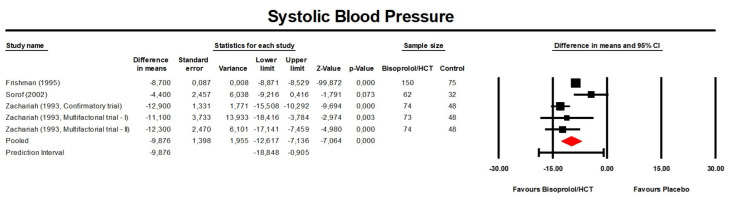
Forest plot displaying mean differences and 95% confidence intervals (CI) for the effect of bisoprolol/hydrochlorothiazide on systolic blood pressure compared to the placebo. CI, confidence interval; HCT, hydrochlorothiazide.

Meta-analysis of available data showed that treatment with bisoprolol/hydrochlorothiazide significantly reduced SBP compared to the control (placebo or another anti-hypertensive treatment) (MD: −8.35 mmHg, 95% CI [−11.44, −5.25] mmHg, *p* < 0.001; I^2^ = 83.2%; Number of trials = 5; Number of individuals = 844; Figure 2) and placebo (MD: −9.88 mmHg, 95% CI [−12.62, −7.14] mmHg, *p* < 0.001; I^2^ = 74.2%; number of trials = 4; Number of individuals = 684; Figure 3).

These findings were robust in the sensitivity analyses (Figures S1 and S2).

Visual inspection of the Begg’s funnel plot revealed a slight asymmetry, suggesting a potential publication bias for the effect of bisoprolol/hydrochlorothiazide on SBP (Figure S3). This asymmetry was imputed to a potentially missing study on the right side of the funnel plot, which increased the estimated effect size to −7.2 (95% CI: −10.48, −3.93). However, Egger’s linear regression and Begg’s rank correlation did not confirm the presence of publication bias (*p* > 0.05). The classic fail-safe N test suggested that 3711 studies with negative results would be needed to bring the estimated effect size on SBP to a non-significant level (*p* > 0.05).

### 3.3. Bisoprolol/Hydrochlorothiazide Effect on DBP

**Figure 4:**
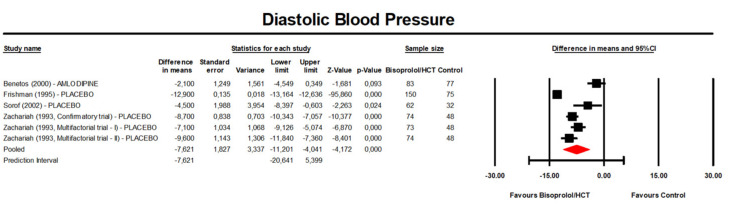
Forest plot displaying mean differences and 95% confidence intervals (CI) for the effect of bisoprolol/hydrochlorothiazide on diastolic blood pressure compared to the control (placebo or another anti-hypertensive treatment). CI, confidence interval; HCT, hydrochlorothiazide.

**Figure 5:**
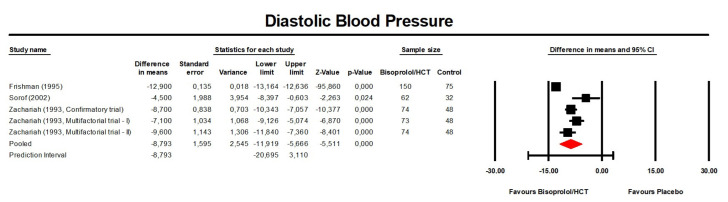
Forest plot displaying mean differences and 95% confidence intervals (CI) for the effect of bisoprolol/hydrochlorothiazide on diastolic blood pressure compared to the control. CI, confidence interval; HCT, hydrochlorothiazide.

Meta-analysis of available data revealed that treatment with bisoprolol/hydrochlorothiazide significantly reduced DBP compared to the control (placebo or another anti-hypertensive treatment) (MD: −7.62 mmHg, 95% CI [−11.20, −4.04] mmHg, *p* < 0.001; I^2^ = 96.6%; number of trials = 5; number of individuals = 844; Figure 4) and placebo (MD: −8.79 mmHg, 95% CI [−11.92, −5.67] mmHg, *p* < 0.001; I^2^ = 94.9%; number of trials = 4; number of individuals = 684; Figure 5).

These findings were robust in the sensitivity analyses (Figures S4 and S5).

Visual inspection of Begg’s funnel plot did not reveal any asymmetry, suggesting no potential publication bias for the effect of bisoprolol/hydrochlorothiazide on DBP (Figure S6). In contrast to Egger’s linear regression, Begg’s rank correlation confirmed the absence of publication bias (Begg’s test, *p* > 0.05; Egger’s test, *p* < 0.001). The classic fail-safe N test suggested that 4091 studies with negative results would be needed to bring the estimated effect size on DBP to a non-significant level (*p* > 0.05).

### 3.4. BP Response Rate after Treatment with Bisoprolol/Hydrochlorothiazide

**Figure 6:**
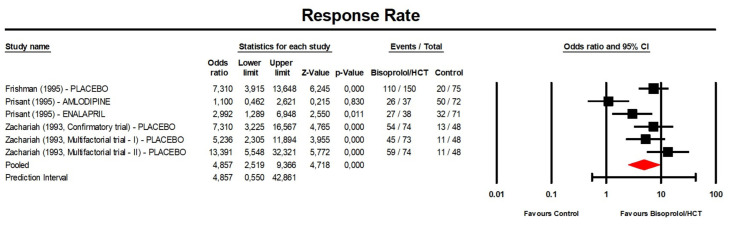
Forest plot displaying the odd ratios and 95% confidence intervals (CI) for the effect of bisoprolol/hydrochlorothiazide on blood pressure response rate compared to the control. CI, confidence interval; HCT, hydrochlorothiazide.

A meta-analysis of the available data showed that the BP response rate after treatment with bisoprolol/hydrochlorothiazide was significantly greater than that of the control (placebo or another anti-hypertensive treatment) (OR: 4.86, 95% CI [2.52, 9.37], *p* < 0.001; I^2^ = 75.2%; number of trials = 4; number of individuals = 808; Figure 6).

This finding was robust in the sensitivity analysis (Figure S7).

### 3.5. BP Control Rate after Treatment with Bisoprolol/Hydrochlorothiazide

**Figure 7:**
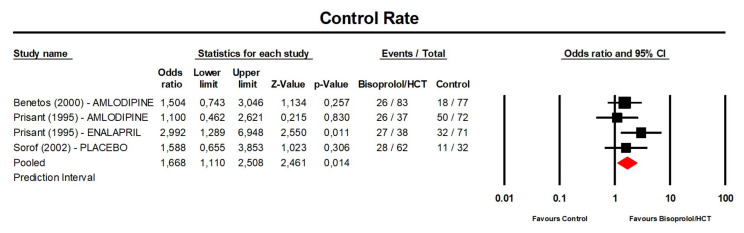
Forest plot displaying the odd ratios and 95% confidence intervals (CI) for the effect of bisoprolol/hydrochlorothiazide on blood pressure control rate compared to the control. CI, confidence interval; HCT, hydrochlorothiazide.

A meta-analysis of the available data showed that the BP control rate after treatment with bisoprolol/hydrochlorothiazide was significantly greater than that of the control (placebo or another anti-hypertensive treatment) (OR: 1.67, 95%CI [1.11, 2.51], *p* = 0.014; I^2^ = 0%; number of trials = 3; number of individuals = 472; Figure 7).

This finding was robust in the sensitivity analysis (Figure S8).

### 3.6. Safety Analysis

Compared to the control, treatment with bisoprolol/hydrochlorothiazide was associated with a reduced risk of AE and peripheral edema. The results of the safety analyses are presented in Table 3. Forest plots displaying the ORs and 95% CI are included in the Supplementary Materials (Figures S9–S22).

## 4. Discussion

Pathophysiological studies have confirmed that the fixed combination of low doses of the two synergistic medications may be beneficial to counter the activation of counter-regulatory hormones [34,35]. Recently, the attention to the use of new-generation beta-blockers in the management of hypertension has been again raised by the recent ESH statement supporting its potential use as a first-step anti-hypertensive drug, based on an attentive revision of outcome trials [36].

Bisoprolol is an FDA- and EMA-approved drug for the management of high BP as well as the low fixed doses of bisoprolol and hydrochlorothiazide [17,37]. From a pharmacological point of view, the addition of hydrochlorothiazide to bisoprolol would increase its anti-hypertensive efficacy, while bisoprolol could compensate the eventual increase of heart rate secondary to the volume depletion related to the diuretic action [38]. In this context, our meta-analysis of the available trials has quantified these effects. The magnitude of the bisoprolol/hydrochlorothiazide BP-lowering effect observed in our meta-analysis (−8.35 mmHg for SBP and −7.62 mmHg for DBP; *p* < 0.001) is clinically meaningful and translated to a substantial reduction in the risk of cardiovascular events. Thus, the significant reductions in both SBP and DBP observed with bisoprolol/hydrochlorothiazide compared to control or placebo suggest that this combination therapy is highly effective in lowering blood pressure.

In a randomized control trial conducted by Frishman et al., more favorable results were observed in terms of BP control, resulting in a significant reduction in SBP levels by 15.8 mmHg and DBP by 12.6 mmHg compared to using bisoprolol or a thiazide diuretic alone [39]. In another study conducted by Luna et al., a follow-up of 106 patients for a minimum of eight weeks after receiving combined therapy of bisoprolol and a low-dose thiazide diuretic yielded a significant reduction in SBP from 157 mmHg to 137 mmHg, as well as a decrease in DBP from 98 mmHg to 87 mmHg [40].

In comparison to our analysis, the previously mentioned trials had a follow-up period ranging from 8 to 12 weeks. However, in our analysis, the follow-up duration was a minimum of 4 weeks and a maximum of 17 weeks [29,30,31,32,33], which may explain the difference in BP improvement as well as the higher medication dosage used [30,40].

The benefits of this combination were demonstrated due to synergistic effects in reducing BP and heart rate, but the extent of the pressure reduction was higher with combined therapy than bisoprolol alone. This is likely due to lower activation of the renin system, neurohumoral inhibition with beta-blockers, and better endothelial function compared to hydrochlorothiazide alone [41]. This is reflected on mortality reduction and decreased hospitalizations in patients with heart failure, patients with reduced systolic function [42], first line therapy for chronic coronary artery disease [43], and survival benefits for post-MI patients [44].

The higher BP response rate observed with bisoprolol/hydrochlorothiazide compared to control is another important finding, since BP response rate is an indicator of how fast the individual patient’s response is in achieving target BP levels. It is important to note that patients with resistant hypertension or patients who require aggressive blood pressure control need to have a quick response rate. The desired therapeutic response ranged between 60 and 71% of cases [30,40,45]. In comparison, the response rates were 69% in the amlodipine arm and 45% for in the enalapril arm [46].

Beta-blockers, such as bisoprolol, are commonly prescribed for various cardiovascular conditions, including uncomplicated hypertension. However, one of the potential side effects of beta-blockers is the development of peripheral edema, which is characterized by the accumulation of fluid in the extremities and is secondary to the reactive stimulation of RAAS [47,48]. This can be bothersome for patients and may even warrant discontinuation of the medication. The addition of hydrochlorothiazide, a thiazide diuretic, to the treatment regimen appears to address this concern. Thiazide diuretics work by increasing the excretion of sodium and water from the body, which helps reduce fluid retention and subsequently lower the risk of edema [49]. By combining bisoprolol with hydrochlorothiazide, the risk of peripheral edema is mitigated, improving patient comfort and compliance with the medication.

The negative impact of thiazide diuretics or beta-blockers on libido or erectile dysfunction are known with higher doses, but we did not detect them in our analysis. Previous reports suggested male sexual dysfunction is not negligible and patient selection is important [46,50].

There are several limitations to consider in our meta-analysis. Firstly, studies included may have focused primarily on the low-dose combination of bisoprolol and hydrochlorothiazide, and the results may not be directly applicable to higher doses. Secondly, our analysis was based on the available literature data from the included studies, and individual patient characteristics and comorbidities were not accounted for. It also needs to be acknowledged that statistical heterogeneity was high for the explored outcomes. For this reason, the studies’ findings were combined using a random-effect model. When heterogeneity is very high and between-study variation dominates, random-effect meta-analyses weight studies nearly equally, regardless of sample sizes, yielding a meta-analytic summary close to the more easily calculated arithmetic mean of the individual study results [25]. Sub-group analysis was performed in an attempt to reduce heterogeneity for the primary outcomes, and sensitivity analysis was used to verify the robustness of the main results. Then, these limitations do not diminish the relevance of our findings, which reaffirm the safety and efficiency of prescribing low-dose bisoprolol/hydrochlorothiazide combinations in stage I and II hypertension.

## Figures and Tables

**Table 1:** Main characteristics of the clinical studies testing the effect of treatment with bisoprolol and hydrochlorothiazide on the considered clinical outcomes.

First Author et al., Year	Location	Follow-Up	Main Inclusion Criteria and Underlying Disease	Study Group	Enrolled Participants (N)	Age (Years; Mean ± SD)	Male Sex (%)
Benetos et al., 2000 [29]	France	12 weeks	60 years of age uncomplicated isolated hypertension	Bisoprolol 2.5 mg/hydrochlorothiazide 6.25 mg	84	72 ± 7	29
				Amlodipine 5 mg	80	73 ± 7.1	47
Frishman et al., 1995 [30]	United States of America	4 weeks	21 years of age stage I or II systemic hypertension	Bisoprolol 5 mg/hydrochlorothiazide 6.25 mg	150	NA	57
				Placebo	75	NA	64
Prisant et al., 1995 [31]	United States of America	17 weeks	≥ 21 years of age sitting DBP between 95 and 114 mmHg	Bisoprolol/hydrochlorothiazide 6.25 mg	75	53 ± 11.5	64
				Amlodipine	72	53 ± 10.1	64
				Enalapril	71	55 ± 9.6	63
Sorof et al., 2002 [32]	United States of America	12 weeks	6–17 years of age SBP and/or DBP above the 95th percentile	Bisoprolol/hydrochlorothiazide 6.25 mg	62	13.8 ± 3.1	56
				Placebo	32	14 ± 2.7	59
Zachariah et al. (1993)–Confirmatory trial [33]	United States of America	12 weeks	21 years of age sitting DBP between 95 and 115 mmHg	Bisoprolol 5 mg/hydrochlorothiazide 6.25 mg	NA	NA	NA
				Placebo			
Zachariah et al. (1993)–Multifactorial trial [33]				Bisoprolol/hydrochlorothiazide 6.25 mg			
				Placebo			

DBP, diastolic blood pressure; N, number of enrolled individuals; NA, not available; SBP, systolic blood pressure; SD, standard deviation.

**Table 2:** Quality of bias assessment of the included studies according to the Cochrane guidelines.

First Author et al., Year	Randomization Process	Comment for Randomization Process	Deviations from Intended Interventions	Comment for Deviations from Intended Interventions	Missing Outcome Data	Comment for Missing Outcome Data	Measurement of the Outcome	Comment for Measurement of the Outcome	Selection of the Reported Results	Comment for Selection of the Reported Results	Overall Bias	Comment for Overall Bias
Benetos et al., 2000 [29]	Low risk of bias	Adequate randomization methods No allocation concealment	Low risk of bias	Double blinding was maintained in the treatment phase of the study by dispensing the investigational products in identical capsules	Low risk of bias	Reasons for attrition reported	Low risk of bias	Members of the study site and the patients were unaware of the treatment assignment during the treatment phase of the study	Low risk of bias	Outcomes reported as specified in methods	Low risk of bias	The study is judged to be at low risk of bias for all domains
Frishman et al., 1995 [30]	Low risk of bias	Adequate randomization methods	Low risk of bias	Double blinding was maintained in the treatment phase of the study by dispensing the investigational products in identical capsules	Low risk of bias	Reasons for attrition reported	Low risk of bias	Members of the study site and the patients were unaware of the treatment assignment during the treatment phase of the study	Low risk of bias	Outcomes reported as specified in methods	Low risk of bias	The study is judged to be at low risk of bias for all domains
Prisant et al., 1995 [31]	Low risk of bias	No allocation concealment	Low risk of bias	Blinding was maintained by dispensing the investigational products in identical capsules	Low risk of bias	Reasons for attrition reported	Low risk of bias	Members of the study site and the patients were unaware of the treatment assignment	Low risk of bias	Outcomes reported as specified in methods	Low risk of bias	The study is judged to be at low risk of bias for all domains
Sorof et al., 2002 [32]	Low risk of bias	Adequate randomization methods	Low risk of bias	Double blinding was maintained in the treatment phase of the study by dispensing the investigational products in identical capsules	Low risk of bias	Reasons for attrition reported	Low risk of bias	Members of the study site and the patients were unaware of the treatment assignment during the treatment phase of the study	Low risk of bias	Outcomes reported as specified in methods	Low risk of bias	The study is judged to be at low risk of bias for all domains
Zachariah et al., 1993 [33]	Low risk of bias	No allocation concealment	Low risk of bias	Double blinding was maintained in the treatment phase of the studies by dispensing the investigational products in identical capsules	Low risk of bias	Reasons for attrition reported	Low risk of bias	Members of the study site and the patients were unaware of the treatment assignment during the treatment phase of the study	Low risk of bias	Outcomes reported as specified in methods	Low risk of bias	The study is judged to be at low risk of bias for all domains

**Table 3:** Adverse events occurred in at least two clinical studies.

Adverse Events	Number of Studies Included in the Analysis	Number of Individuals Considered for the Analysis	Odd Ratio	95% Confidence Interval		*p*-Value	I^2^
				Lower Limit	Upper Limit		
Any Adverse Event	4	697	0.69	0.48	0.99	0.043	26%
Headache	4	697	0.7	0.36	1.36	0.294	32%
Insomnia	4	968	1	0.31	3.17	0.994	0%
Dizziness	3	808	1.62	0.55	4.79	0.386	0%
Fatigue	4	968	1.82	0.83	4.01	0.138	0%
Bradycardia	4	968	2.61	0.68	10.1	0.164	0%
Hypokalemia	2	449	1.57	0.16	15.07	0.697	0%
Cough	2	443	0.69	0.12	3.95	0.681	0%
Dyspnea	2	443	1.31	0.22	7.68	0.767	4%
Nausea	3	603	0.37	0.06	2.19	0.273	0%
Diarrhea	2	443	0.42	0.05	3.44	0.416	0%
Peripheral Edema	3	603	0.21	0.05	0.87	0.031	0%
Decrease or Loss of Libido	3	808	0.72	0.15	3.43	0.681	0%
Impotence	4	968	2.18	0.6	7.93	0.237	0%

## Data Availability

Data supporting the findings of this analysis are available from the authors with the permission of the University of Bologna. The results from the present study have been presented during the 32nd European Meeting on Hypertension and Cardiovascular Protection (Milan, June 2023).

## Supplementary Materials

The following supporting information can be downloaded at: https://www.mdpi.com/article/10.3390/jcm13154572/s1, Figure S1: Plots showing the leave-one-out sensitivity analysis for the effect on SBP of bisoprolol/hydrochlorothiazide compared to control (placebo or another anti-hypertensive treatment); Figure S2: Plots showing the leave-one-out sensitivity analysis for the effect on SBP of bisoprolol/hydrochlorothiazide compared to placebo; Figure S3: Funnel plot detailing publication bias for the effect on SBP of bisoprolol/hydrochlorothiazide compared to control (placebo or another anti-hypertensive treatment); Figure S4: Plots showing the leave-one-out sensitivity analysis for the effect on DBP of bisoprolol/hydrochlorothiazide compared to control (placebo or another anti-hypertensive treatment); Figure S5: Plots showing the leave-one-out sensitivity analysis for the effect on DBP of bisoprolol/hydrochlorothiazide compared to placebo; Figure S6: Funnel plot detailing publication bias for the effect on DBP of bisoprolol/hydrochlorothiazide compared to control (placebo or another anti-hypertensive treatment); Figure S7: Plots showing the leave-one-out sensitivity analysis for the effect on BP response rate of bisoprolol/hydrochlorothiazide compared to control; Figure S8: Plots showing the leave-one-out sensitivity analysis for the effect on BP control rate of bisoprolol/hydrochlorothiazide compared to control; Figure S9: Forest plot displaying the odd ratios and 95% confidence intervals for the risk of any adverse event following treatment with bisoprolol/hydrochlorothiazide compared to control; Figure S10: Forest plot displaying the odd ratios and 95% confidence intervals for the risk of headache following treatment with Bisoprolol/Hydrochlorothiazide compared to control; Figure S11: Forest plot displaying the odd ratios and 95% confidence intervals for the risk of insomnia following treatment with bisoprolol/hydrochlorothiazide compared to control; Figure S12: Forest plot displaying the odd ratios and 95% confidence intervals for the risk of dizziness following treatment with bisoprolol/hydrochlorothiazide compared to control; Figure S13: Forest plot displaying the odd ratios and 95% confidence intervals for the risk of fatigue following treatment with Bisoprolol/Hydrochlorothiazide compared to control; Figure S14: Forest plot displaying the odd ratios and 95% confidence intervals for the risk of bradycardia following treatment with bisoprolol/hydrochlorothiazide compared to control; Figure S15: Forest plot displaying the odd ratios and 95% confidence intervals for the risk of hypokalemia following treatment with Bisoprolol/Hydrochlorothiazide compared to control; Figure S16: Forest plot displaying the odd ratios and 95% confidence intervals for the risk of cough following treatment with bisoprolol/hydrochlorothiazide compared to control; Figure S17: Forest plot displaying the odd ratios and 95% confidence intervals for the risk of dyspnea following treatment with bisoprolol/hydrochlorothiazide compared to control; Figure S18: Forest plot displaying the odd ratios and 95% confidence intervals for the risk of nausea following treatment with bisoprolol/hydrochlorothiazide compared to control; Figure S19: Forest plot displaying the odd ratios and 95% confidence intervals for the risk of diarrhea following treatment with bisoprolol/hydrochlorothiazide compared to control; Figure S20: Forest plot displaying the odd ratios and 95% confidence intervals for the risk of peripheral edema following treatment with bisoprolol/hydrochlorothiazide compared to control; Figure S21: Forest plot displaying the odd ratios and 95% confidence intervals for the risk of decrease or loss of libido following treatment with bisoprolol/hydrochlorothiazide compared to control; Figure S22: Forest plot displaying the odd ratios and 95% confidence intervals for the risk of impotence following treatment with bisoprolol/hydrochlorothiazide compared to control.

## Appendix A. Studies Excluded from the Meta-Analysis after Assessment

- Not controlled clinical studies (*n* = 3) [34,40,51]
- Clinical trials containing overlapping individuals with other studies (*n* = 4) [45,52,53,54]
- Testing bisoprolol/hydrochlorothiazide combination versus another anti-hypertensive combination with bisoprolol or hydrochlorothiazide (*n* = 2) [55,56]
- Lack of a control group receiving placebo or a BP-lowering control treatment different from bisoprolol or hydrochlorothiazide (*n* = 1) [57]
