# Supplementary material for: Efficacy and Safety of Low-Dose Bisoprolol/Hydrochlorothiazide Combination for the Treatment of Hypertension: A Systematic Review and Meta-Analysis
Source: J Clin Med. 2024 Aug 5;13(15):4572. doi: 10.3390/jcm13154572 (PMC11313031; doi:10.3390/jcm13154572)
Supplement: Supplementary file 1 [file jcm-13-04572-s001.zip › jcm-3092545-supplementary.pdf]

SUPPLEMENTARY MATERIALS

Figure S1 **–** Plots showing the leave-one-out sensitivity analysis for the effect on SBP of Bisoprolol / Hydrochlorothiazide compared to control (placebo or another anti-hypertensive treatment).

Systolic Blood Pressure

Statistics with study removed

Study name	Point	Standard error	Variance	Lower limit	Upper limit	Z-Value	p-Value
Benetos (2000) - AMLODIPINE	-9,876	1,398	1,955	-12,617	-7,136	-7,064	0,000
Frishman (1995) - PLACEBO	-8,188	2,730	7,452	-13,538	-2,838	-3,000	0,003
Sorof (2002) - PLACEBO	-9,039	1,758	3,090	-12,485	-5,594	-5,142	0,000
Zachariah (1993, Confirmatory trial) - PLACEBO	-7,198	1,908	3,639	-10,937	-3,459	-3,773	0,000
Zachariah (1993, Multifactorial trial - I) - PLACEBO	-8,013	1,726	2,980	-11,396	-4,629	-4,642	0,000
Zachariah (1993, Multifactorial trial - II) - PLACEBO	-7,627	1,785	3,185	-11,125	-4,129	-4,273	0,000
	-8,345	1,581	2,498	-11,443	-5,247	-5,279	0,000

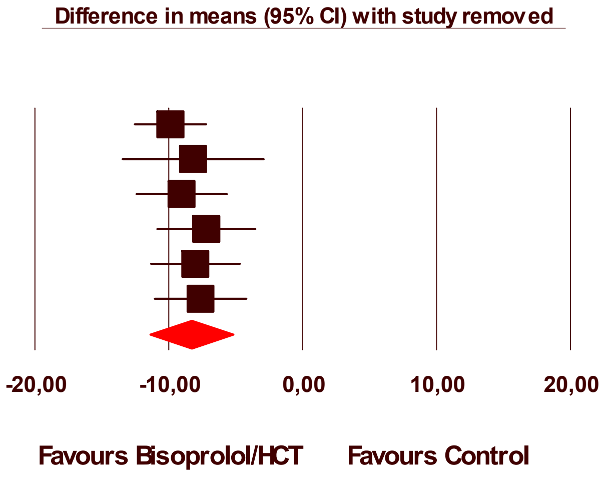

CI= Confidence Interval; HCT= Hydrochlorothiazide.

Figure S2 – Plots showing the leave-one-out sensitivity analysis for the effect on SBP of Bisoprolol / Hydrochlorothiazide compared to placebo.

Systolic Blood Pressure

Study name	Statistics with study removed							Difference in means (95% CI) with study removed
	Point	Standard error	Variance	Lower limit	Upper limit	Z-Value	p-Value	
Frishman (1995)	-10,323	2,079	4,323	-14,398	-6,247	-4,965	0,000	
Sorof (2002)	-10,935	1,521	2,312	-13,916	-7,955	-7,192	0,000	
Zachariah (1993, Confirmatory trial)	-8,806	1,313	1,723	-11,379	-6,233	-6,708	0,000	
Zachariah (1993, Multifactorial trial - I)	-9,736	1,545	2,388	-12,765	-6,707	-6,300	0,000	
Zachariah (1993, Multifactorial trial - II)	-9,400	1,577	2,486	-12,490	-6,310	-5,962	0,000	
	-9,876	1,398	1,955	-12,617	-7,136	-7,064	0,000	

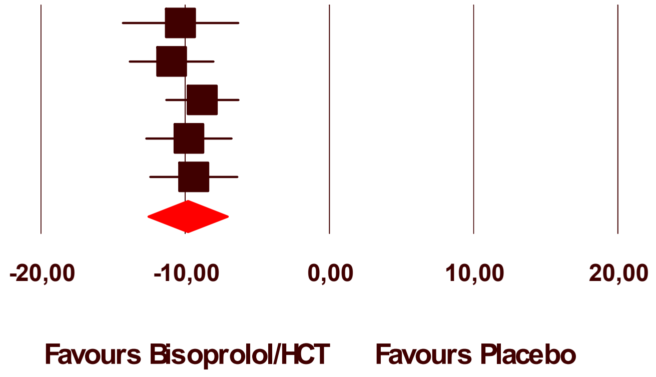

CI= Confidence Interval; HCT= Hydrochlorothiazide.

***Figure S3* –** Funnel plot detailing publication bias for the effect on SBP of Bisoprolol / Hydrochlorothiazide compared to control (placebo or another anti-hypertensive treatment).

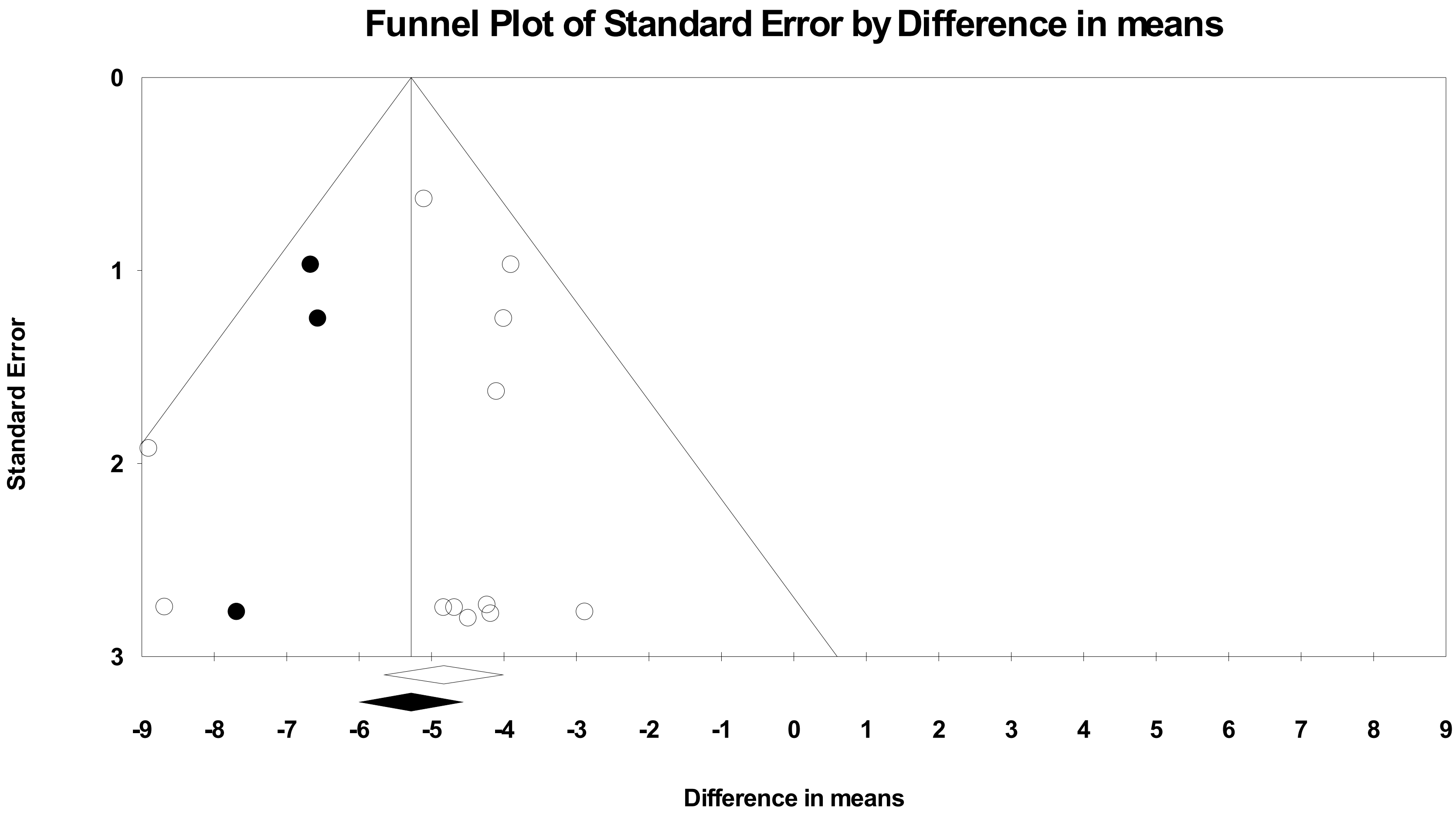

***Figure S4* –** Plots showing the leave-one-out sensitivity analysis for the effect on DBP of Bisoprolol / Hydrochlorothiazide compared to control (placebo or another anti-hypertensive treatment).

Diastolic Blood Pressure

Study name	Statistics for each study						
	Difference in means	Standard error	Variance	Lower limit	Upper limit	Z-Value	p-Value
Frishman (1995)	-12,900	0,135	0,018	-13,164	-12,636	-95,860	0,000
Sorof (2002)	-4,500	1,988	3,954	-8,397	-0,603	-2,263	0,024
Zachariah (1993, Confirmatory trial)	-8,700	0,838	0,703	-10,343	-7,057	-10,377	0,000
Zachariah (1993, Multifactorial trial - I)	-7,100	1,034	1,068	-9,126	-5,074	-6,870	0,000
Zachariah (1993, Multifactorial trial - II)	-9,600	1,143	1,306	-11,840	-7,360	-8,401	0,000
	-8,793	1,595	2,545	-11,919	-5,666	-5,511	0,000

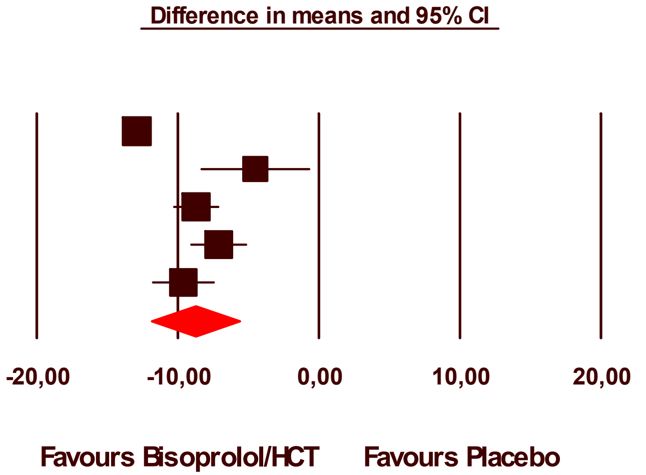

CI= Confidence Interval; HCT= Hydrochlorothiazide.

Figure S5 – Plots showing the leave-one-out sensitivity analysis for the effect on DBP of Bisoprolol / Hydrochlorothiazide compared to placebo.

Diastolic Blood Pressure

Study name	Statistics with study removed						
	Point	Standard error	Variance	Lower limit	Upper limit	Z-Value	p-Value
Frishman (1995)	-7,927	0,847	0,717	-9,587	-6,266	-9,358	0,000
Sorof (2002)	-9,654	1,619	2,620	-12,827	-6,482	-5,964	0,000
Zachariah (1993, Confirmatory trial)	-8,773	1,957	3,830	-12,609	-4,937	-4,483	0,000
Zachariah (1993, Multifactorial trial –[illegible])	-9,266	1,666	2,776	-12,531	-6,000	-5,561	0,000
Zachariah (1993, Multifactorial trial –[illegible])	-8,553	1,924	3,701	-12,323	-4,782	-4,446	0,000
	-8,793	1,595	2,545	-11,919	-5,666	-5,511	0,000

Difference in means (95% CI) with study removed

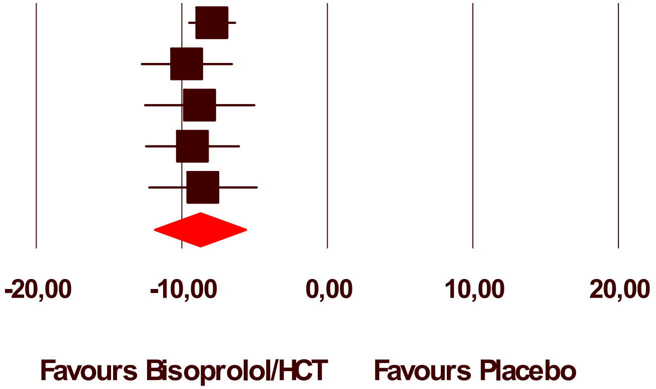

CI= Confidence Interval; HCT= Hydrochlorothiazide.

Figure S6 – Funnel plot detailing publication bias for the effect on DBP of Bisoprolol / Hydrochlorothiazide compared to control (placebo or another anti-hypertensive treatment).

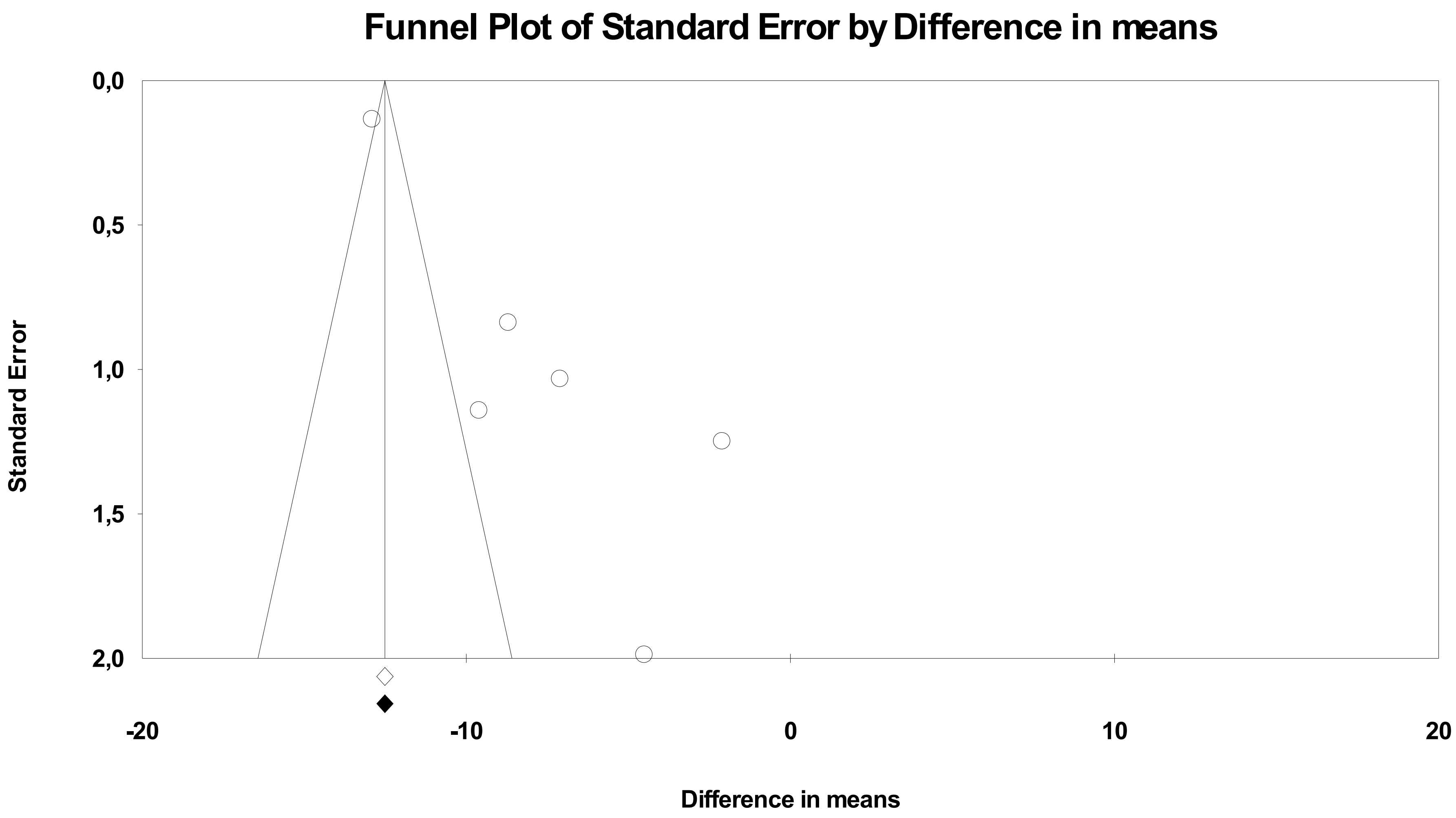

Figure S7 – Plots showing the leave-one-out sensitivity analysis for the effect on BP response rate of Bisoprolol / Hydrochlorothiazide compared to control.

Response Rate

Study name	Statistics with study removed					Odds ratio (95% CI) with study removed
	Point	Lower limit	Upper limit	Z-Value	p-Value	
Frishman (1995) - PLACEBO	4,424	1,967	9,950	3,596	0,000	
Prisant (1995) - AMLODIPINE	6,465	4,150	10,071	8,252	0,000	
Prisant (1995) - ENALAPRIL	5,333	2,491	11,417	4,309	0,000	
Zachariah (1993, Confirmatory trial) - PLACEBO	4,469	2,041	9,783	3,745	0,000	
Zachariah (1993, Multifactorial trial - I) - PLACEBO	4,773	2,142	10,634	3,824	0,000	
Zachariah (1993, Multifactorial trial - II) - PLACEBO	4,020	2,043	7,912	4,028	0,000	
	4,857	2,519	9,366	4,718	0,000	

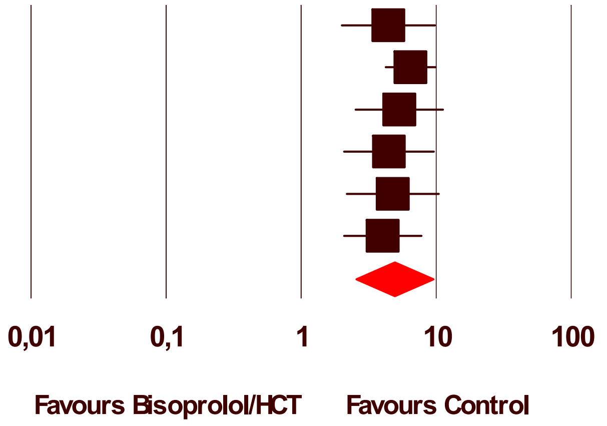

CI= Confidence Interval; HCT= Hydrochlorothiazide.

Figure S8 – Plots showing the leave-one-out sensitivity analysis for the effect on BP control rate of Bisoprolol / Hydrochlorothiazide compared to control.

Control Rate

Study name	Statistics with study removed					Odds ratio (95%CI) with study removed
	Point	Lower limit	Upper limit	Z-Value	p-Value	
Benetos (2000) - AMLODIPINE	1,757	1,066	2,895	2,212	0,027	
Prisant (1995) - AMLODIPINE	1,877	1,183	2,978	2,673	0,008	
Prisant (1995) - ENALAPRIL	1,395	0,876	2,223	1,402	0,161	
Sorof (2002) - PLACEBO	1,690	1,068	2,676	2,241	0,025	
	1,668	1,110	2,508	2,461	0,014	

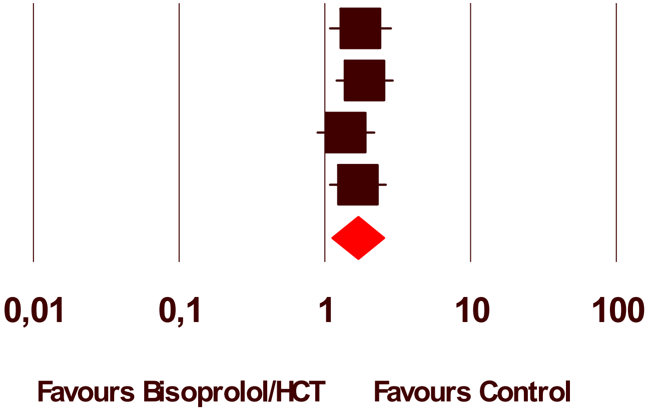

CI= Confidence Interval; HCT= Hydrochlorothiazide.

Figure S9 – Forest plot displaying the odd ratios and 95% confidence intervals for the risk of any adverse event following treatment with Bisoprolol / Hydrochlorothiazide compared to control.

Any Adverse Event

Study name	Statistics for each study				
	Odds ratio	Lower limit	Upper limit	Z-Value	p-Value
Benetos (2000) - AMLODIPINE	0,928	0,494	1,745	-0,232	0,817
Frishman (1995) - PLACEBO	1,354	0,522	3,509	0,624	0,533
Prisant (1995) - AMLODIPINE	0,564	0,241	1,320	-1,320	0,187
Prisant (1995) - ENALAPRIL	0,461	0,199	1,069	-1,806	0,071
Sorof (2002) - PLACEBO	0,379	0,148	0,974	-2,015	0,044
	0,687	0,477	0,989	-2,021	0,043

Odds ratio and 95%CI

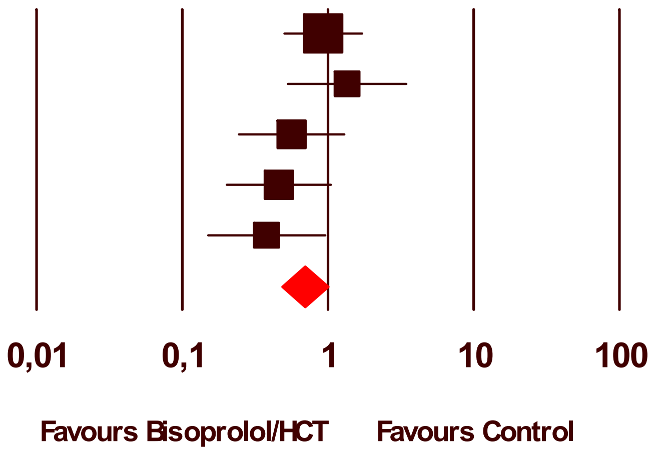

CI= Confidence Interval; HCT= Hydrochlorothiazide.

Figure S10 **–** Forest plot displaying the odd ratios and 95% confidence intervals for the risk of headache following treatment with Bisoprolol / Hydrochlorothiazide compared to control.

Headache

Study name	Odds ratio	Lower limit	Upper limit	Z-Value	p-Value
	**Statistics for each study**				
Benetos (2000) - AMLODIPINE	0,122	0,015	1,015	-1,946	0,052
Frishman (1995) - PLACEBO	0,103	0,005	2,202	-1,455	0,146
Prisant (1995) - AMLODIPINE	2,087	0,392	11,123	0,862	0,389
Prisant (1995) - ENALAPRIL	0,879	0,211	3,660	-0,177	0,860
Sorof (2002) - PLACEBO	0,765	0,299	1,958	-0,558	0,577
	0,703	0,364	1,357	-1,050	0,294

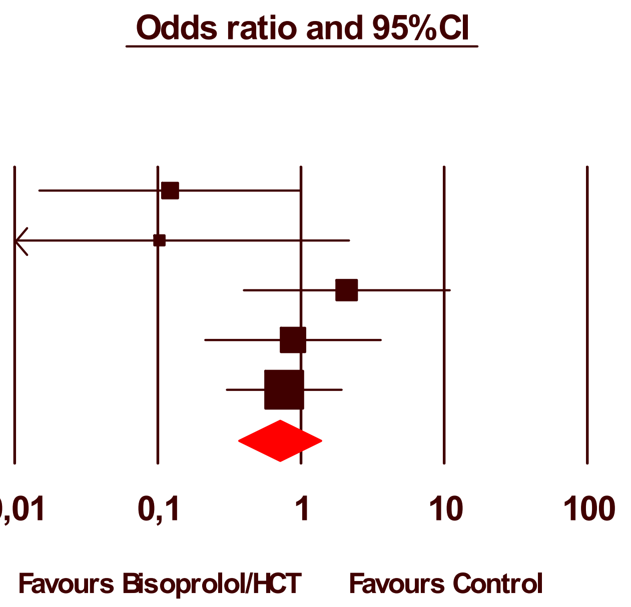

CI= Confidence Interval; HCT= Hydrochlorothiazide.

***Figure S11* –** Forest plot displaying the odd ratios and 95% confidence intervals for the risk of insomnia following treatment with Bisoprolol / Hydrochlorothiazide compared to control.

Insomnia

Study name	Odds ratio	Lower limit	Upper limit	Z-Value	p-Value
Benetos (2000) - AMLODIPINE	4,755	0,225	100,621	1,001	0,317
Frishman (1995) - PLACEBO	1,084	0,088	13,386	0,063	0,950
Prisant (1995) - AMLODIPINE	1,000	0,019	53,760	0,000	1,000
Prisant (1995) - ENALAPRIL	1,000	0,019	52,423	0,000	1,000
Zachariah (1993) - PLACEBO	0,595	0,111	3,198	-0,605	0,545
	0,995	0,313	3,168	-0,008	0,994

Statistics for each study

Odds ratio and 95%CI

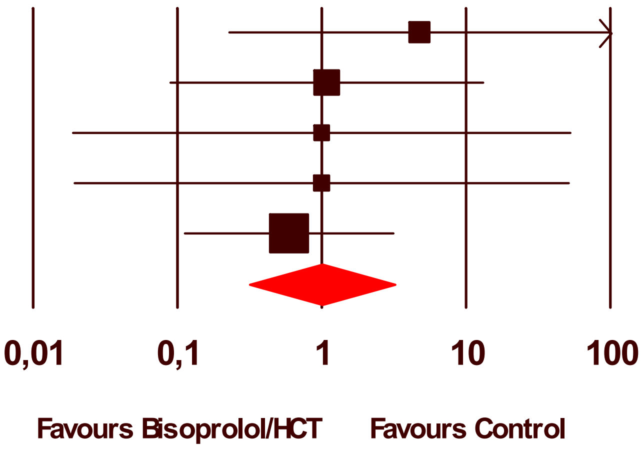

CI= Confidence Interval; HCT= Hydrochlorothiazide.

Figure S12 **–** Forest plot displaying the odd ratios and 95% confidence intervals for the risk of dizziness following treatment with Bisoprolol / Hydrochlorothiazide compared to control.

Dizziness

Study name	Statistics for each study				
	Odds ratio	Lower limit	Upper limit	Z-Value	p-Value
Frishman (1995) - PLACEBO	2,634	0,271	25,607	0,835	0,404
Prisant (1995) - AMLODIPINE	3,398	0,103	112,588	0,685	0,493
Prisant (1995) - ENALAPRIL	0,158	0,006	4,476	-1,081	0,280
Zachariah (1993) - PLACEBO	1,803	0,428	7,603	0,803	0,422
	1,616	0,545	4,789	0,866	0,386

Odds ratio and 95%CI

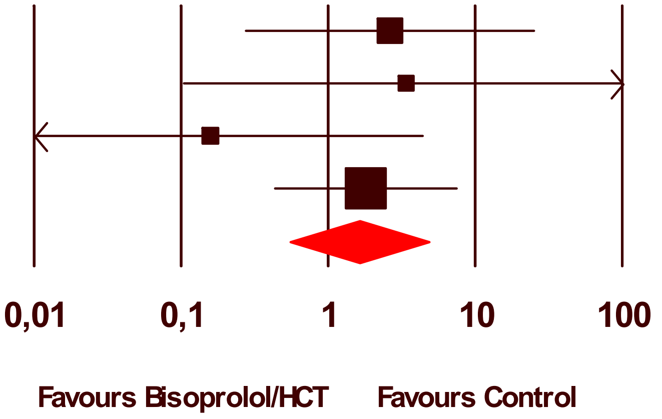

CI= Confidence Interval; HCT= Hydrochlorothiazide.

Figure S13 – Forest plot displaying the odd ratios and 95% confidence intervals for the risk of fatigue following treatment with Bisoprolol / Hydrochlorothiazide compared to control.

Fatigue

Study name	Odds ratio	Lower limit	Upper limit	Z-Value	p-Value
	**Statistics for each study**				
Benetos (2000) - AMLODIPINE	3,454	0,695	17,168	1,515	0,130
Frishman (1995) - PLACEBO	2,111	0,208	21,428	0,632	0,527
Prisant (1995) - AMLODIPINE	0,825	0,140	4,839	-0,214	0,831
Prisant (1995) - ENALAPRIL	1,702	0,231	12,535	0,522	0,602
Zachariah (1993) - PLACEBO	1,788	0,407	7,865	0,769	0,442
	1,819	0,825	4,009	1,484	0,138

Odds ratio and 95%CI

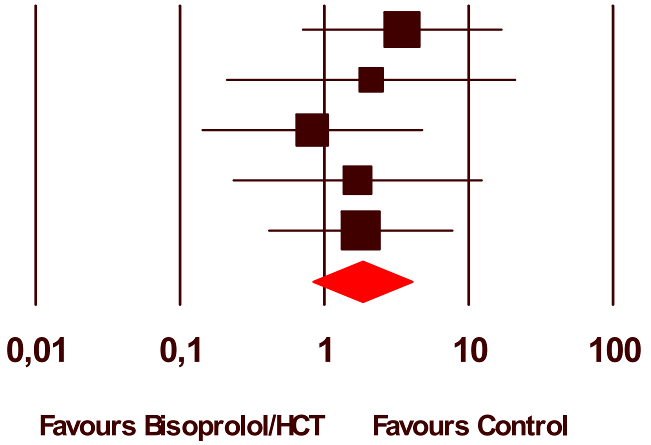

CI= Confidence Interval; HCT= Hydrochlorothiazide.

***Figure S14* –** Forest plot displaying the odd ratios and 95% confidence intervals for the risk of bradycardia following treatment with Bisoprolol / Hydrochlorothiazide compared to control.

Bradycardia

Study name	Odds ratio	Lower limit	Upper limit	Z-Value	p-Value
Benetos (2000) - AMLODIPINE	8,774	0,465	165,702	1,449	0,147
Frishman (1995) - PLACEBO	1,413	0,055	36,114	0,209	0,834
Prisant (1995) - AMLODIPINE	3,398	0,103	112,588	0,685	0,493
Prisant (1995) - ENALAPRIL	3,301	0,100	108,582	0,670	0,503
Zachariah (1993) - PLACEBO	1,288	0,116	14,280	0,206	0,836
	2,614	0,676	10,098	1,393	0,164

Odds ratio and 95%CI

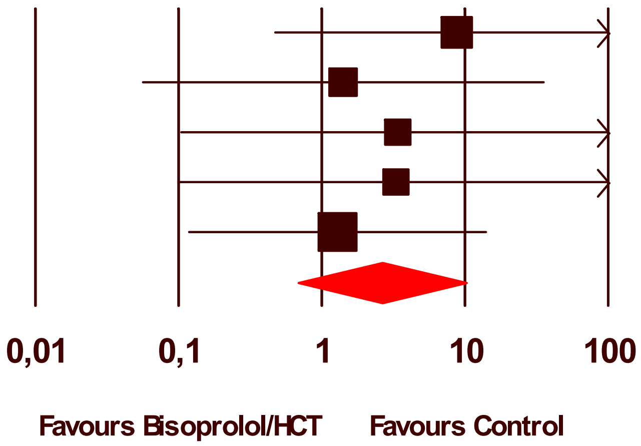

CI= Confidence Interval; HCT= Hydrochlorothiazide.

***Figure S15* –** Forest plot displaying the odd ratios and 95% confidence intervals for the risk of hypokalemia following treatment with Bisoprolol / Hydrochlorothiazide compared to control.

Hypokalemia

Study name	Odds ratio	Lower limit	Upper limit	Z-Value	p-Value
Frishman (1995) - PLACEBO	1,566	0,064	38,407	0,275	0,783
Zachariah (1993) - PLACEBO	1,569	0,064	38,558	0,276	0,783
	1,568	0,163	15,071	0,389	0,697

Statistics for each study

Odds ratio and 95%CI

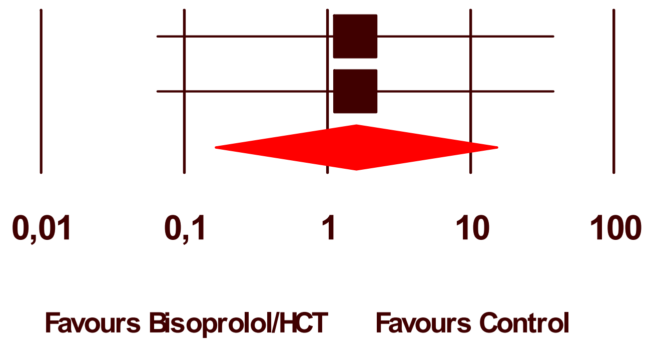

CI= Confidence Interval; HCT= Hydrochlorothiazide.

***Figure S16* –** Forest plot displaying the odd ratios and 95% confidence intervals for the risk of cough following treatment with Bisoprolol / Hydrochlorothiazide compared to control.

Cough

Study name	Odds ratio	Lower limit	Upper limit	Z-Value	p-Value
	Statistics for each study				
Frishman (1995) - PLACEBO	3,426	0,173	67,747	0,809	0,419
Prisant (1995) - AMLODIPINE	0,275	0,014	5,495	-0,845	0,398
Prisant (1995) - ENALAPRIL	0,343	0,016	7,229	-0,689	0,491
	0,694	0,122	3,945	-0,412	0,681

Odds ratio and 95%CI

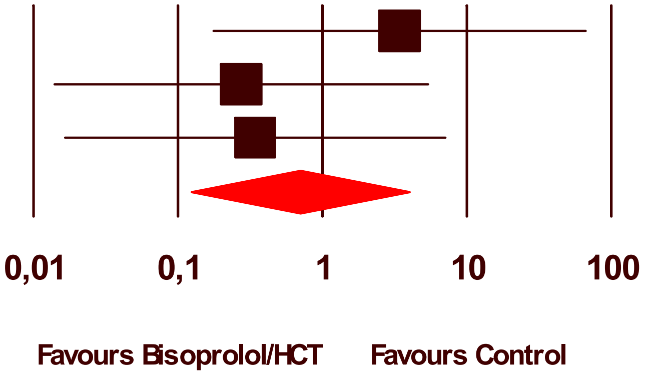

CI= Confidence Interval; HCT= Hydrochlorothiazide.

***Figure S17* –** Forest plot displaying the odd ratios and 95% confidence intervals for the risk of dyspnea following treatment with Bisoprolol / Hydrochlorothiazide compared to control.

Dyspnea

Study name	Odds ratio	Lower limit	Upper limit	Z-Value	p-Value
Frishman (1995) - PLACEBO	0,177	0,007	4,525	-1,047	0,295
Prisant (1995) - AMLODIPINE	3,062	0,154	61,060	0,733	0,464
Prisant (1995) - ENALAPRIL	3,062	0,154	60,877	0,734	0,463
	1,307	0,222	7,682	0,296	0,767

Odds ratio and 95%CI

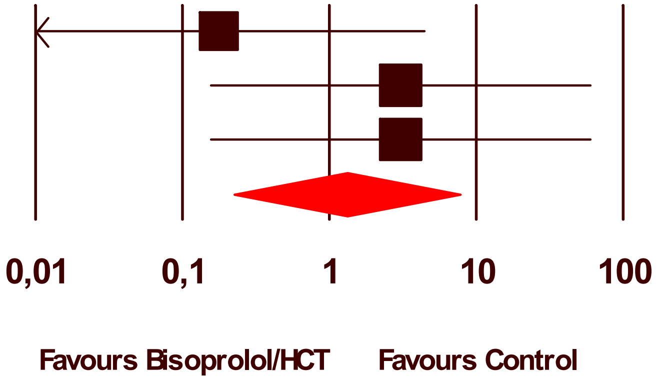

CI= Confidence Interval; HCT= Hydrochlorothiazide.

Figure S18 **–** Forest plot displaying the odd ratios and 95% confidence intervals for the risk of nausea following treatment with Bisoprolol / Hydrochlorothiazide compared to control.

Nausea

Study name	Odds ratio	Lower limit	Upper limit	Z-Value	p-Value
Benetos (2000) - AMLODIPINE	0,127	0,006	2,508	-1,355	0,175
Frishman (1995) - PLACEBO	1,413	0,055	36,114	0,209	0,834
Prisant (1995) - ENALAPRIL	0,343	0,016	7,229	-0,689	0,491
	0,369	0,062	2,190	-1,097	0,273

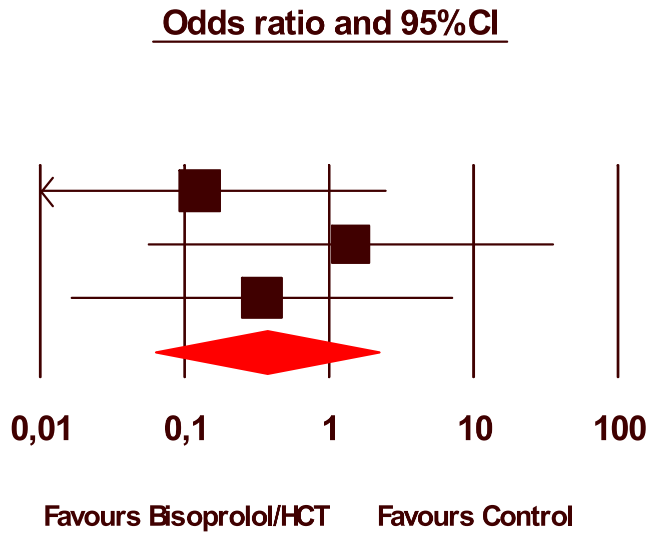

CI= Confidence Interval; HCT= Hydrochlorothiazide.

***Figure S19* –** Forest plot displaying the odd ratios and 95% confidence intervals for the risk of diarrhea following treatment with Bisoprolol / Hydrochlorothiazide compared to control.

Diarrhea

Study name	Odds ratio	Lower limit	Upper limit	Z-Value	p-Value
Frishman (1995) - PLACEBO	0,497	0,026	9,354	-0,467	0,641
Prisant (1995) - ENALAPRIL	0,343	0,016	7,229	-0,689	0,491
	0,416	0,050	3,443	-0,814	0,416

Odds ratio and 95%CI

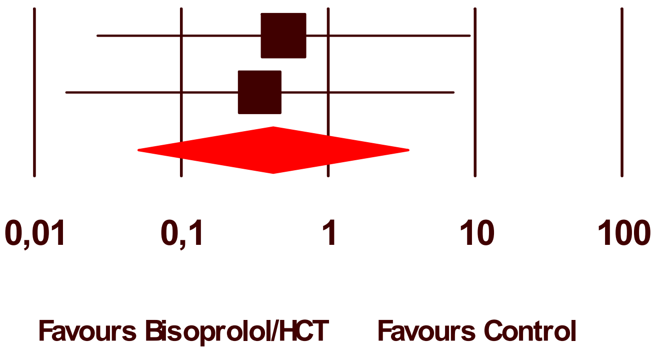

CI= Confidence Interval; HCT= Hydrochlorothiazide.

***Figure S20* –** Forest plot displaying the odd ratios and 95% confidence intervals for the risk of peripheral edema following treatment with Bisoprolol / Hydrochlorothiazide compared to control.

Peripheral edema

Study name	Statistics for each study				
	Odds ratio	Lower limit	Upper limit	Z-Value	p-Value
Benetos (2000) - AMLODIPINE	0,122	0,015	1,015	-1,946	0,052
Frishman (1995) - PLACEBO	1,413	0,055	36,114	0,209	0,834
Prisant (1995) - AMLODIPINE	0,091	0,003	2,536	-1,412	0,158
Prisant (1995) - ENALAPRIL	0,242	0,008	7,329	-0,815	0,415
	0,209	0,050	0,866	-2,158	0,031

Odds ratio and 95%CI

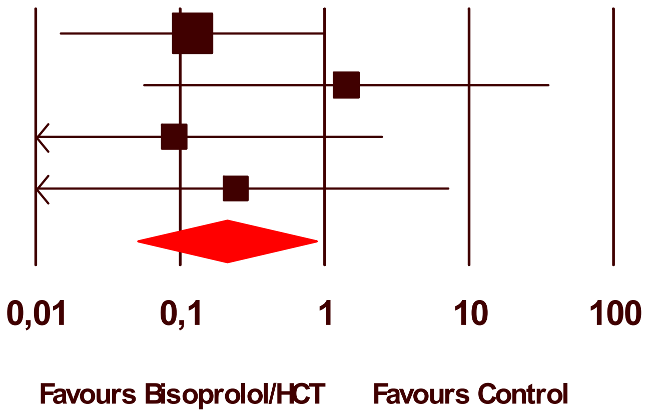

CI= Confidence Interval; HCT= Hydrochlorothiazide.

Figure S21 – Forest plot displaying the odd ratios and 95% confidence intervals for the risk of decrease or loss of libido following treatment with Bisoprolol / Hydrochlorothiazide compared to control.

Decrease or loss of libido

Study name	Statistics for each study					Odds ratio and 95%CI
	Odds ratio	Lower limit	Upper limit	Z-Value	p-Value	
Frishman (1995) - PLACEBO	0,177	0,007	4,525	-1,047	0,295	
Prisant (1995) - AMLODIPINE	3,398	0,103	112,588	0,685	0,493	
Prisant (1995) - ENALAPRIL	3,301	0,100	108,582	0,670	0,503	
Zachariah (1993) - PLACEBO	0,331	0,025	4,327	-0,843	0,399	
	0,720	0,151	3,434	-0,412	0,681	

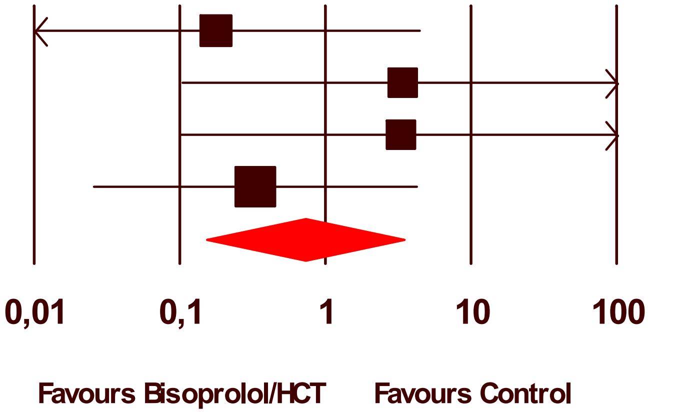

CI= Confidence Interval; HCT= Hydrochlorothiazide.

***Figure S22* –** Forest plot displaying the odd ratios and 95% confidence intervals for the risk of impotence following treatment with Bisoprolol / Hydrochlorothiazide compared to control.

Impotence

Study name	Statistics for each study					Odds ratio and 95%CI
	Odds ratio	Lower limit	Upper limit	Z-Value	p-Value	
Benetos (2000) - AMLODIPINE	2,818	0,113	70,223	0,632	0,528	
Frishman (1995) - PLACEBO	1,413	0,055	36,114	0,209	0,834	
Prisant (1995) - AMLODIPINE	3,062	0,154	61,060	0,733	0,464	
Prisant (1995) - ENALAPRIL	3,062	0,154	60,877	0,734	0,463	
Zachariah (1993) - PLACEBO	1,578	0,153	16,240	0,383	0,701	
	2,180	0,599	7,932	1,183	0,237	

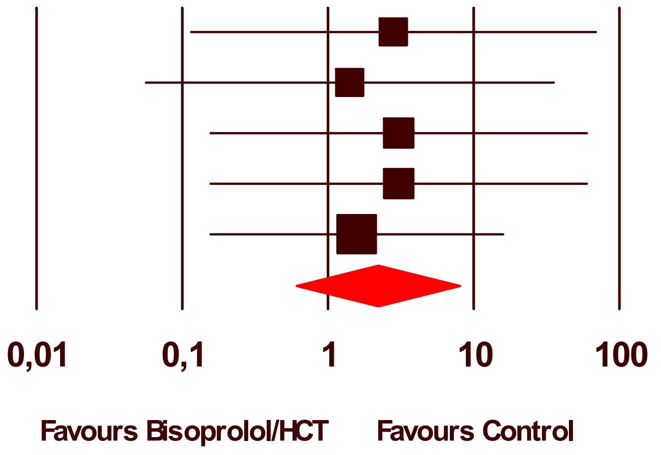

CI= Confidence Interval; HCT= Hydrochlorothiazide.
